# Adapting the Time-Domain Synthetic Aperture Focusing Technique (T-SAFT) to Laser Ultrasonics for Imaging the Subsurface Defects

**DOI:** 10.3390/s23198036

**Published:** 2023-09-22

**Authors:** Sundara Subramanian Karuppasamy, Che-Hua Yang

**Affiliations:** Graduate Institute of Manufacturing Technology, National Taipei University of Technology, Taipei 10608, Taiwan; chyang@ntut.edu.tw

**Keywords:** laser ultrasonics, laser-induced phased array, defect imaging, non-destructive testing, time-domain synthetic aperture focusing technique (T-SAFT), structural health monitoring

## Abstract

Traditional ultrasonic testing uses a single probe or phased array probe to investigate and visualize defects by adapting certain imaging algorithms. The time-domain synthetic aperture focusing technique (T-SAFT) is an imaging algorithm that employs a single probe to scan along the test specimen in various positions, to generate inspection images with better resolution. Both the T-SAFT and phased array probes are contact methods with limited bandwidth. This work aims to combine the advantages of the T-SAFT and phased array in a noncontact way with the aid of laser ultrasonics. Here, a pulsed laser beam is employed to generate ultrasonic waves in both thermoelastic and ablation regimes, whereas the laser Doppler vibrometer is used to acquire the generated signals. These two lasers are focused on the test specimen and, to avoid the plasma and crater influence in the ablation regime, the transmission beam and reception beam are separated by 5 mm. By moving the test specimen with a step size of 0.5 mm, a 1D linear phased array (41 and 43 elements) with a pitch of 0.5 mm was synthesized, and three side-drilled holes (Ø 8 mm—thermoelastic regime, Ø 10 mm and Ø 2 mm—ablation regime) were introduced for inspection. The A-scan data obtained from these elements were processed via the T-SAFT algorithm to generate the inspection images in various grid sizes. The results showed that the defect reflections obtained in the ablation regime have better visibility than those from the thermoelastic regime. This is due to the high-amplitude signals obtained in the ablation regime, which pave the way for enhancing the pixel intensity of each grid. Moreover, the separation distance (5 mm) does not have any significant effect on the defect location during the reconstruction process.

## 1. Introduction

Aluminum alloys are among the predominantly used alloys in most applications. An aluminum alloy is often termed a structural alloy and possesses excellent characteristics, which pave the way for its implementation in fabricating structures where light weight and a high strength-to-weight ratio are the key factors. It consists of a great amount of aluminum, along with other elements, such as silicon, magnesium, manganese, zinc, etc. Alloying these elements with aluminum affords this alloy outstanding properties, such as greater strength, significant resistance against corrosion, low cost, a high degree of processability, better formability, and so on. These characteristics make this alloy a prominent structural material in fabricating the components of marine, aerospace, civil, and nuclear structures [1,2,3]. Based on the composition of alloying elements, this alloy can be categorized into many series, from the 1xxx series to the 8xxx series. Each series has different characteristics, which can be adopted for various applications. Of these series, alloy 6082 T6 is mostly preferred in fabricating aerospace, civil, and marine structures. This alloy has greater strength compared to other alloys belonging to the 6xxx series and is used in a wide range of applications [4,5].

Since this alloy contributes most in terms of fabricating components that work in crucial environments, there is a need to monitor the structural integrity of those components to avoid failures. The formation of defects, such as leak holes, cracks, and pores, serves as a root cause for the lack of structural integrity. To ensure the safety and structural integrity of these components, several non-destructive techniques (NDTs) can be implemented, which will assess the defects in a non-intrusive way. Some of the most commonly used NDTs are the visual NDT, eddy current NDT, magnetic flux leakage NDT, magnetic particle NDT, ultrasonic NDT, and so on [6,7].

Among the above-mentioned techniques, ultrasonic testing is one of the most prominent techniques used for detecting defects. This technique uses ultrasonic (sound) waves that have a higher frequency than the audible limit of humans. This kind of testing uses a transmitter–receiver pair and an imaging tool for examining the test surfaces. The transmitter is said to generate ultrasonic waves, which travel through the inspecting media and are received by the receiver. By transmitting and receiving these waves, the defect information can be seen in the imaging tool as signals [8]. The major advantage is that since this method works under the propagation of ultrasonic waves, both surface and subsurface defects can be examined with great accuracy. Other than this, these waves can penetrate to great depths, the flaws can be detected with high precision, the types of flaws can be imaged by implementing various signal processing techniques, and so on [9].

In most cases, a single transducer probe is used to inspect the parts, and the time-domain signal (A-scan) received by the probe is analyzed to examine the reflected echo from the defect. This reflected echo will show the position of the defect based on the time-of-flight calculation. The major limitation of using a single transducer probe is that the defect location is predicted based on the reflected echo with a single time-domain signal, and the geometry of the defect cannot be visualized. Also, this A-scan databased inspection is reported to provide limited flexibility during the inspection of complex shapes [10]. On the other hand, the transducer array consists of many transducers, which will pave the way for a greater number of transmitter–receiver combinations followed by a vast amount of information about the defect [11]. These signals are processed with the help of signal processing and imaging techniques, which will convert them into test images for identifying the defect’s geometries and locations in the test specimen.

In a transducer array, the distance between each transducer element (pitch) determines the array configuration. Based on this pitch, the transducer array can be categorized into two configurations, namely sparse array and phased array [12]. In sparse array, the transducer elements are arranged randomly with irregular pitch. This type of array has several drawbacks as follows: (i) the elements need to be mounted over a great area to perform the inspection, (ii) uneven beam-forming and beam-steering properties, (iii) quite tricky for complex structures, (iv) the received signals contain coherent noises, which are produced from the edges and other geometries in the inspected specimen, (v) low contrast in inspected images, (vi) need for baseline comparison, and so on [13,14]. These limitations can be overcome by using the phased array configuration. A phased array probe contains a series of transducer elements that are arranged in a predefined fashion with regular pitch. This type of array has many advantages, such as (i) optimal beam-forming and beam-steering characteristics, (ii) suitable to inspect complex shapes as it does not need more transducers, and the beam can be steered at the required angles for inspecting complex specimens, (iii) the coherent noises are comparatively lower than the sparse array, (iv) there is no need for any baseline comparison techniques, (v) a wide range of imaging algorithms can be adapted to generate the inspection images, (vi) there is good contrast in the inspected images, and so on [15,16].

Conventional ultrasonic inspection uses imaging techniques such as triangulation [17] and tomographic reconstruction [18]. The inspection images generated through these techniques lack resolution since the number of elements is limited. Another technique is termed delay and sum beam-forming, where time delays are added to each signal and summed together to generate the inspection images. These time delays are added at the transmitter source to develop the images. All three techniques are reported to produce low-resolution images with a limited number of elements [19].

The time-domain synthetic aperture focusing technique (T-SAFT) is a post-processing technique, which is applied to a single transducer probe to generate high-resolution inspection images. This technique consists of single probe, which is said to generate and receive ultrasonic signals at various positions on the test specimen [20]. The test specimen serves as the imaging grid and the time taken for the ultrasonic signal from the probe to every grid point is calculated and mapped with its corresponding amplitude. This process is repeated until all amplitudes are summed up from every probe position for every grid to generate inspection images. Mostly, this technique is adapted to improve the resolution of the inspection images, and also the defect’s geometries can be visualized [21,22]. The reliability of this technique depends on the transducer position, grid structure, and bandwidth of the probe. Both sparse and phased array probe inspections are said to be contact methods (the probe should be in contact with the test specimen), which use a great number of elements with limited bandwidth, are not good for rough surfaces, and have a high probability of inspection errors where temperature fluctuation is a key factor.

Therefore, researchers are working on optical methods with which to perform inspection. Laser ultrasonic testing is one among those methods, which involves a pulsed laser to generate ultrasonic waves and a laser interferometer to receive waves. This type of inspection has major advantages over the traditional probes, such as (i) contactless inspection, (ii) greater sensitivity with excellent bandwidth, (iii) couplant-free inspection, (iv) multimodality, and so on [23,24]. In this technique, ultrasonic pulses are generated in two regimes, namely thermoelastic and ablation regimes [25]. The difference between these two regimes lies in the laser power and amplitude of the signals.

Zhang X et al. [26] established a fully noncontact system based on laser ultrasound in medical imaging and reported that their system would have a notable influence on the implementation of laser ultrasound in the clinical industry. Selim H et al. [27] proposed an inspection system based on the SAFT principle. Their study included a pulsed laser beam to generate waves that scanned over the test surface, which were produced by conventional ultrasonic transducers. Yoon T et al. [28] analyzed the influence of acoustic velocity during the image reconstruction process by using the laser ultrasonic T-SAFT and concluded that accurate acoustic velocity extraction could enhance the visibility in reconstructed images. Ying KN et al. [29] investigated the directivity patterns of multi-modes, which were generated via pulsed laser beam, and adapted these multi-modes to generate inspection images via the T-SAFT algorithm. Pei C et al. [30] proposed a fully noncontact system to evaluate defects based on a laser-induced fiber phased-array setup.

This work aims to investigate subsurface defects in an aluminum block (AA 6082 T6) by combining laser ultrasonics with the T-SAFT principle via a laser-induced linear phased-array system. A fully noncontact system is proposed, which combines a pulsed laser to generate waves in both thermoelastic and ablation regimes with a laser Doppler vibrometer (LDV) used to receive ultrasonic waves. The pulsed laser and the LDV beams are focused on the aluminum block (test specimen) as the point source. These two beams are separated by 5 mm and the test specimen is mounted on the translation stage of the stepper motor. With simultaneous movement of the motor with a step size of 0.5 mm, a 1D laser-induced linear phased array (41 and 43 elements) with a pitch of 0.5 mm is synthesized. The time-domain (A-scan) signals acquired from these elements are processed using the T-SAFT. As a result, inspection images are generated at various pixel resolutions to visualize the defects.

## 2. Materials and Methods

### 2.1. Laser Ultrasound Generation

**Figure 1:**
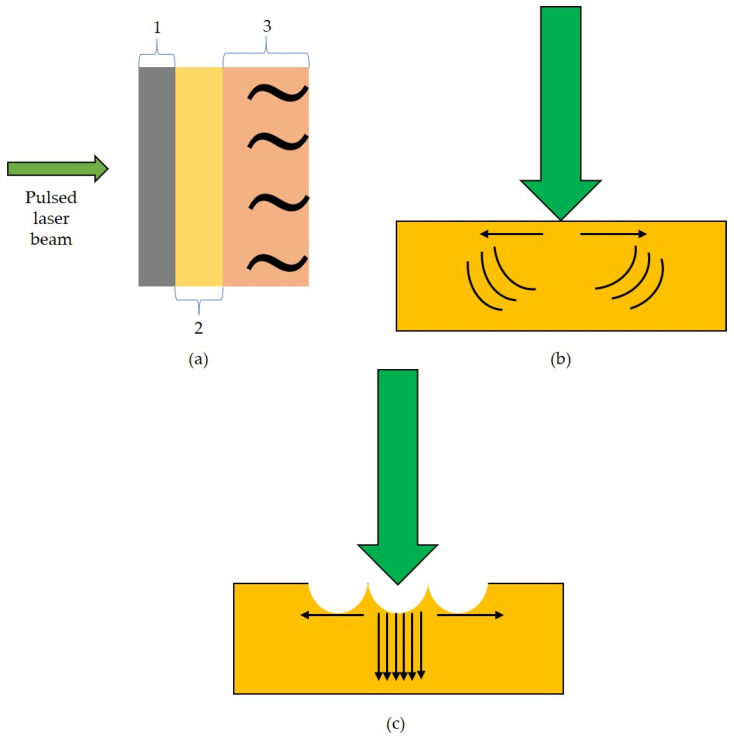
(**a**) Schematic illustration of laser ultrasound generation (1—absorption of electromagnetic radiation, 2—thermal expansion, 3—ultrasonic wave emission); (**b**) thermoelastic regime—direction of arrows represents the propagation of ultrasonic waves, whereas the curves represent the momentum transfer [36]; (**c**) ablation regime—downward arrows represent the direction of momentum transfer along with the propagation of strong signals, which are normal to the test surface [36].

As discussed earlier, the laser ultrasound technique is a contact-free technique to generate ultrasonic waves. Usually, a pulsed laser is involved in the generation of ultrasonic waves. The pulsed beam heats the surface, which thermally expands to generate ultrasonic waves (Figure 1a). Based on the laser power, ultrasound generation can be performed in two regimes: (i) thermoelastic regime, and (ii) ablation regime [31].

#### 2.1.1. Thermoelastic Regime

This regime is considered the common way to generate ultrasound. With low laser power, the pulsed laser beam is focused on the test specimen. This beam is absorbed to a particular depth by the specimen, which is reported to undergo thermal expansion. During this expansion, the heated region in the sample delivers stress that serves as the source for generating ultrasonic waves [32]. The generation of ultrasound through the thermoelastic regime is schematically illustrated in Figure 1b. In this regime, there is no crater formation at the focused spot, and the ultrasonic signals generated under this regime contain lower-amplitude signals than those in the ablation regime.

#### 2.1.2. Ablation Regime

The ablation regime generates ultrasonic waves in higher amplitudes. This regime is similar to the thermoelastic regime but it involves a high-power laser beam to generate ultrasonic waves. In metals, when the applied laser power is above the threshold limit, the sample’s surface is reported to vaporize since the surface cannot sustain the applied laser power. In addition, this power is able to ionize the surrounding air, thereby creating a plasma plume that surrounds the beam spot on the test surface. As a result, a crater is formed that is surrounded by the dark plasma plume [33,34]. Figure 1c represents the laser ablation phenomenon.

### 2.2. Laser Ultrasound Detection

For noncontact detection of ultrasound, a laser Doppler vibrometer (LDV) is employed in this work. This vibrometer senses the ultrasonic wave by employing the superposition principle. It consists of a laser beam, which is split by a beam splitter into two beams termed measurement and reference beams. The measurement beam is focused on the test specimen, which is under stress or vibration, whereas the reference beam is directed to the photodetector. The backscattered measurement beam is influenced by this stress, which is reported to change the phase and frequency of the backscattered measurement beam. This backscattered beam is superpositioned with the reference beam to generate the output signal [35]. This optical method of detecting the ultrasonic waves is non-invasive and better spatial distribution can be accomplished.

### 2.3. Time-Domain Synthetic Aperture Focusing Technique (T-SAFT)

**Figure 2:**
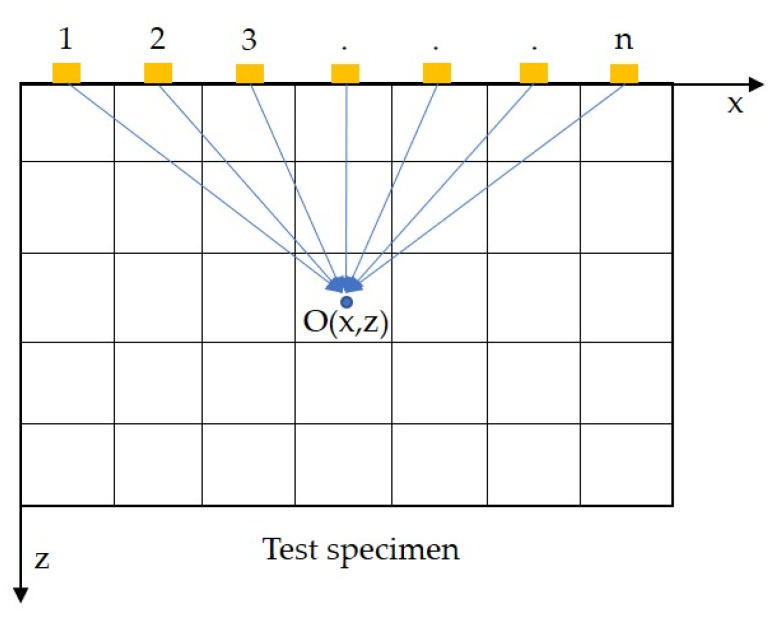
Traditional T-SAFT principle where 1, 2, 3 … *n* denotes the probe position. Here, the probe position is replaced by synthesized 1D laser-induced linear phased array elements.

The time-domain synthetic aperture focusing technique (T-SAFT) is the prominent post-processing technique implemented in traditional ultrasonic testing (single-probe testing). Figure 2 shows a schematic representation of the T-SAFT principle. The test specimen serves as the grid and the aperture (probe) is said to generate and receive ultrasonic signals in various positions along the scanning direction into the specified grid *O*(*x*,*z*). The intensity value at *O*(*x*,*z*) is calculated as the summation of all amplitudes of ultrasonic signals generated from each position in terms of their time-of-flight calculations. The intensity of grid *O*(*x*,*z*) is as follows [37,38]:

(1) $$O\left(x,z\right)=\left|\sum_{k=1}^{N}h_{k}\left(2\frac{\sqrt{{\left(x_{k}-x\right)}^{2}+{\left(z\right)}^{2}}}{c_{l}}\right)\right|$$

where *k* is the probe position, *N* is the number of probe positions along the scanning direction, *x* and *z* are grid coordinates, $c_{l}$ is the longitudinal wave velocity, and *h* is the Hilbert transform operator, which is an optional operator not used in single-probe SAFT inspection [38]. While performing inspections with the phased array probe, researchers used Hilbert transform to process the received A-scan data. This transform shifts the A-scan data in phase by π/2 radians. This phase shifting aids in extracting an accurate envelope of the signal without any loss of data when compared with the other typical filters. Also, this Hilbert envelope provides better visibility in the inspection images during the phased-array probe inspections [39,40].

**Figure 3:**
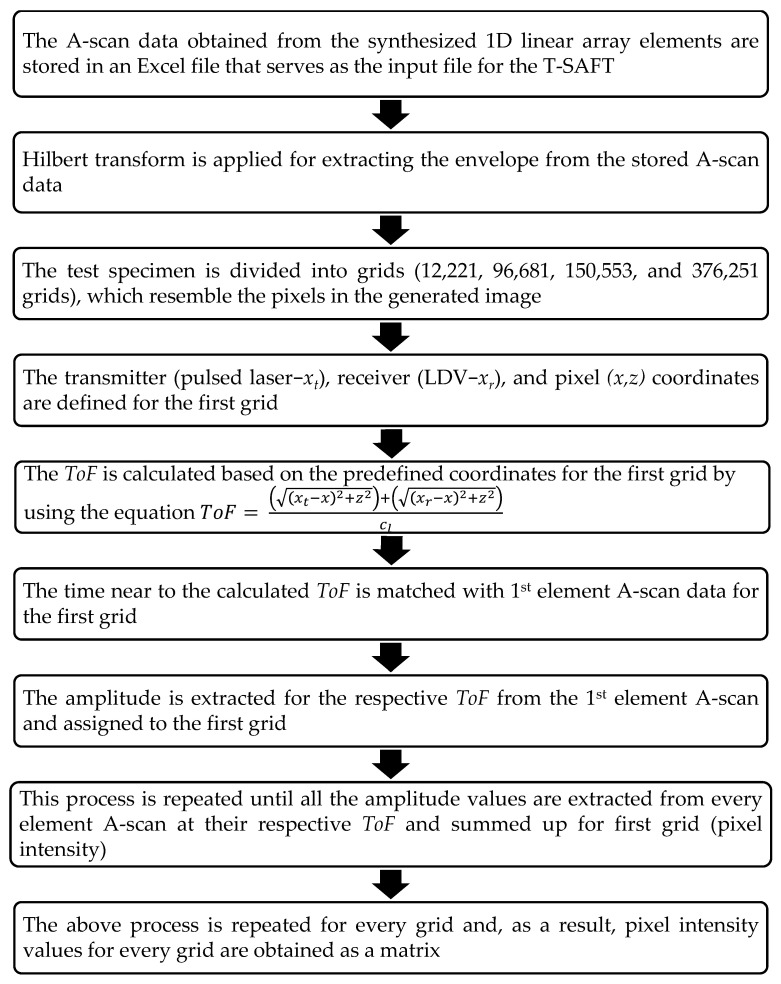
Flowchart of the T-SAFT process [38].

Here, by moving the test specimen, a 1D linear phased array was synthesized by focusing the pulsed laser and Doppler vibrometer laser beams on the test specimen. These two beams were separated by 5 mm. Hence, the above equation was modified as given below for calculating the intensity:

(2) $$O\left(x,z\right)=\left|\sum_{t=1}^{N}\sum_{r=1}^{N}h_{\left(t,r\right)}\left(\frac{\sqrt{{\left(x_{t}-x\right)}^{2}+{\left(z\right)}^{2}}+\sqrt{{\left(x_{r}-x\right)}^{2}+{\left(z\right)}^{2}}}{c_{l}}\right)\right|$$

where *t* and *r* are the pulsed laser beam (transmitter) and vibrometer laser beam (receiver) positions, *h* is the Hilbert transform operator (optional), $c_{l}$ is the longitudinal wave velocity, *x* and *z* represent the pixel (grid) coordinates, and *N* is the number of elements in the synthesized 1D linear array. In Equation (2), the term inside the parentheses is called time of flight (*ToF*), which is defined as the time taken by ultrasonic waves generated from the transmitter (*t*) to reach the pixel (grid) and return to receiver (*r*). In traditional SAFT imaging, a single probe is used to transmit and receive the ultrasonic signal, and the ToF value is thus twice that in Equation (1). However, in this work, the generation laser and detection laser beams were separated by 5 mm; hence, the *ToF* was separated in terms of transmitter and receiver positions in the test specimen. Also, the Hilbert transform is normally used while inspecting the defects with a phased array probe [41]. Here, both raw A-scan data and Hilbert-transformed A-scan data were used to generate inspection images. The flowchart of the T-SAFT process is shown in Figure 3.

### 2.4. Experimental Setup

**Figure 4:**
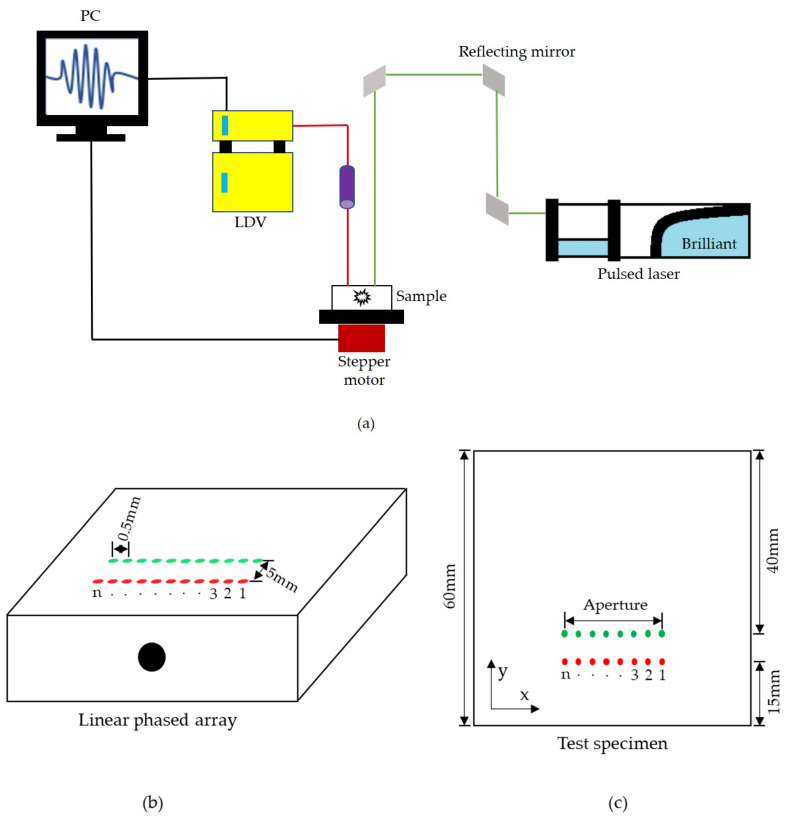
(**a**) Experimental setup; (**b**) synthesized 1D linear array configuration; (**c**) array location in a 2D plane showing the aperture. Numbers 1, 2, 3, … *n* (41 or 43) represent the synthesized linear array elements within the aperture. Green dots represent the generation points (pulsed laser), whereas the red dots represent the reception points (LDV). To inspect 8 mm and 10 mm holes, 41 elements were synthesized with an elemental pitch of 0.5 mm. Hence, the aperture size was reported to be 20.5 mm. On the other hand, for inspecting the three 2 mm holes, 43 elements were synthesized with an elemental pitch of 0.5 mm. Therefore, the aperture size is said to be 21.5 mm.

**Figure 5:**
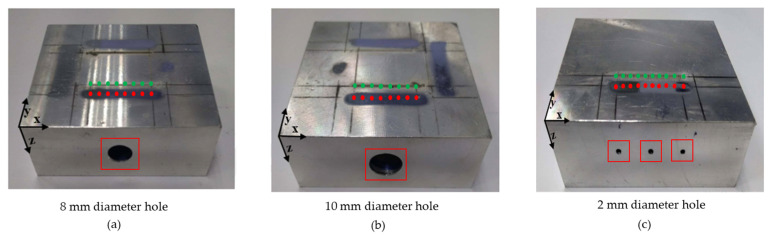
Side drilled holes of 25 mm depth with diameters of (**a**) 8 mm; (**b**) 10 mm; and (**c**) three 2 mm holes (one lies in the center and the other two holes lie at both ends of aperture). The specimen was mirror polished at the reception side to some distance for effective measurement of waves [42]. Green dots represent the generation points (pulsed laser), whereas the red dots represent the reception points (LDV). Red box represents the defects.

Figure 4a shows a schematic illustration of the experimental setup. A Q-switched pulsed Nd: YAG laser (Model: Quantel Brilliant B, Les Ulis, France) with a wavelength of 532 nm was employed to generate ultrasonic waves at a pulse duration of 5 ns. The maximum energy per pulse delivered was 400 mJ. Meanwhile, the generated ultrasonic waves were acquired using a Laser Doppler Vibrometer (vibrometer controller: Polytec OFV 2700, and sensor head: Polytec OFV 511, Irvine, CA, USA) consisting of a continuous helium–neon laser beam (wavelength—633 nm) with bandwidth of 50 kHz–30 MHz and a center frequency of 0.55 MHz. Both pulsed laser (transmitter) and vibrometer (receiver) laser beams were focused on the specimen as the point source. A 1D linear phased array was synthesized by moving the test specimen (mounted on the stepper motor) with a step size of 0.5 mm, which served as the pitch between each element in the synthesized phased array (Figure 4b,c). The LDV provided an efficient measurement of vibrations (signals) when the measurement beam was fully reflected without any disturbances. Better reflectivity of beams could be obtained by mirror polishing the surfaces of metals [42]. On the other hand, retro reflexive foils were used on rough surfaces, where mirror polishing was not possible. Here, the inspection was carried out in aluminum blocks, and the test specimens were mirror polished on the reception side for effective measurement of signals. The inspection was performed in both regimes. The thermoelastic regime did not produce any crater, whereas, in ablation, a crater was formed on the test surface. This crater and the plasma influenced the reflectivity of the measurement beam. Hence, both beams were separated by 5 mm to avoid such influence. This distance was effective for detecting the longitudinal waves [43]. The pulsed laser, vibrometer, and stepper motor were controlled using the LabVIEW program, and from each linear array element, the A-scan data were recorded after averaging 13 times in order to obtain a better SNR. These A-scan data served as inputs for the T-SAFT algorithm, which was programmed by using MATLAB. Three side drilled holes (Figure 5a–c) with diameters of 8 mm, 10 mm, and 2 mm were drilled in the aluminum 6082 T6 blocks.

## 3. Results

### 3.1. Calculation of Maximum and Minimum ToF

Here, the test specimen served as the region of interest (ROI), which was divided into four different grid sizes (pixels) containing 12,221, 96,681, 150,553, and 376,251 pixels. The defect reflections in these four grid sizes with different pixel resolutions were as follows:

(i) 12,221 pixels (121 × 101): x-axis—0.5 mm/pixel and z-axis—0.25 mm/pixel.
(ii) 96,681 pixels (481 × 201): x-axis—0.125 mm/pixel and z-axis—0.125 mm/pixel.
(iii) 150,553 pixels (481 × 313): x-axis—0.125 mm/pixel and z-axis—0.08 mm/pixel.
(iv) 376,251 pixels (751 × 501): x-axis—0.08 mm/pixel and z-axis—0.05 mm/pixel.

In T-SAFT for each grid, the calculated *ToF* was mapped with the nearest time element in the A-scan data for every element in the synthesized linear array; there was a need to make sure that every A-scan data point had the nearest time element for the calculated *ToF*. Hence, the maximum and minimum values of *ToF* required for the four types of grids were calculated, and based on the calculated values, the A-scan data for every element were recorded. For four different grids, the maximum and minimum *ToF* were calculated as follows:

(3) $$Time\;of\;Flight\;(ToF)=\left|\left(\frac{\sqrt{{\left(x_{t}-x\right)}^{2}+{\left(z\right)}^{2}}+\sqrt{{\left(x_{r}-x\right)}^{2}+{\left(z\right)}^{2}}}{c_{l}}\right)\right|$$

where $c_{l}$ is the longitudinal wave velocity that propagates in the aluminum block (test specimen). This wave velocity is constant for every grid and calculated from Young’s modulus, Poisson’s ratio, and the density of the aluminum block, which are listed in Table 1.

The longitudinal wave velocity (Equation (4)) can be calculated from the parameters given in Table 1.

(4) $$Longitudinal\;wave\;velocity,\;c_{l}=\sqrt{\frac{\lambda +2\mu }{\rho }}$$

where

(5) $$\lambda =\frac{E\nu }{\left(1-2\nu \right)(1+\nu )}$$

(6) $$µ=\frac{E}{2(1+\nu )}$$

On substituting the parametric values in Equations (5) and (6), the longitudinal wave velocity of the test specimen (AA 6082 T6) from Equation (4) was found to be 6,230,420 mm/s. By defining the transmitter, receiver, and pixel coordinates along with the $c_{l}$ value, we determined that the minimum and maximum *ToF* values required for all four grids were 0.8 μs and 15.23 μs, respectively. Hence, the A-scan data were recorded up to 15.23 μs.

### 3.2. Imaging in Thermoelastic Regime (Ø 8 mm Hole)

**Figure 6:**
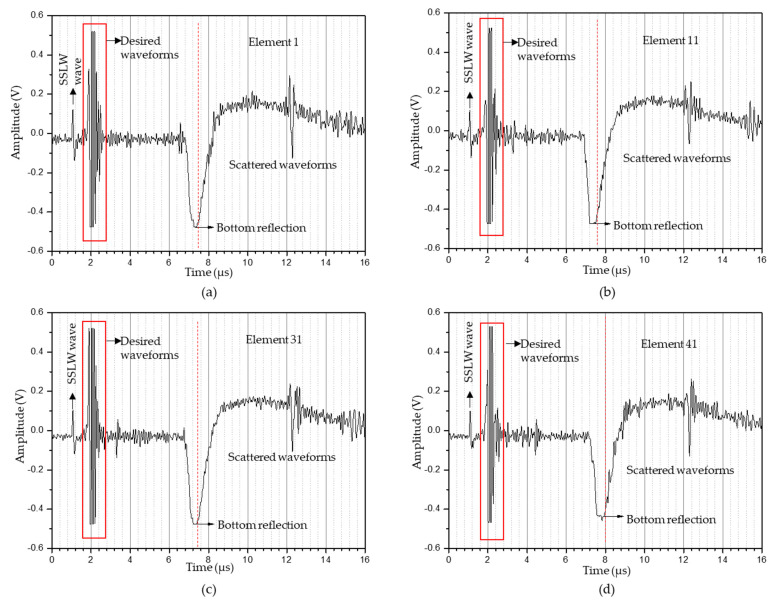
A−scan data obtained in the thermoelastic regime (8 mm hole) from the synthesized linear array elements: (**a**) 1st element; (**b**) 11th element; (**c**) 31st element; (**d**) 41st element. The desired waveforms contain surface acoustic wave (SAW) and defect reflections. The waveforms occurring after the red dotted line (bottom reflection) are scattered waveforms (SSLW—surface skimming longitudinal wave).

The experimental parameters used to generate ultrasonic waves under the thermoelastic regime are listed in Table 2. A 1D linear array of 41 elements with an aperture size of 20.5 mm was synthesized and 41 A-scan data points from those elements were stored in an Excel file used to perform T-SAFT imaging. Figure 6a–d represent the A-scan data recorded from the 1st, 11th, 31st, and 41st LDV elements, respectively.

**Figure 7:**
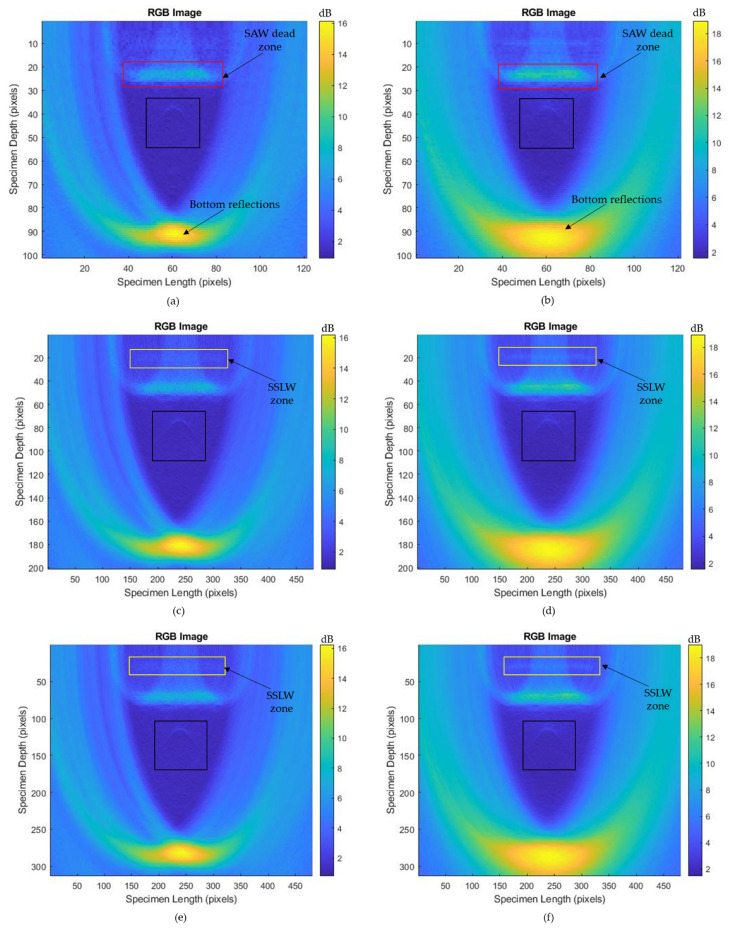

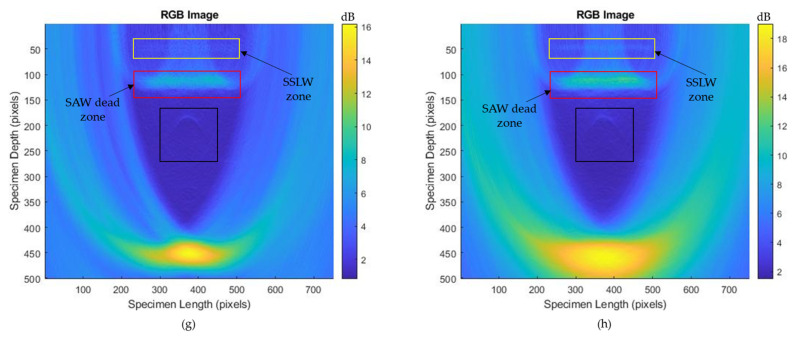
T-SAFT reconstruction images obtained in the thermoelastic regime (8 mm hole) using the raw time traces: (**a**) 12,221 grids, (**c**) 96,681 grids, (**e**) 150,553 grids, (**g**) 376,251 grids; and using the Hilbert-transformed data: (**b**) 12,221 grids, (**d**) 96,681 grids, (**f**) 150,553 grids, (**h**) 376,251 grids. A semicircular arc inside the black box represents the 8 mm hole (top surface). The yellow box represents the surface skimming longitudinal wave (SSLW) zone, whereas the red box represents the surface acoustic wave (SAW) dead zone due to the separation distance of 5 mm between the pulsed laser and LDV.

With these data, the T-SAFT algorithm was performed, and the reconstructed T-SAFT images with the raw time traces (skipping step 2 in the T-SAFT flowchart) and after Hilbert transform at various grid sizes are represented in Figure 7a–h. Figure 7a,c,e,g shows reconstructed T-SAFT images using the raw time traces (acquired A-scan data) obtained from the 41 linear phased-array elements from the grid sizes containing 12,221, 96,681, 150,553, and 376,251 grids. In these figures, the reflection from the 8 mm diameter hole is visualized as the formation of a semicircular arc (top surface of the hole). Moreover, the defect reflections could be enhanced by increasing the number of grids in the ROI (test specimen). Hence, the visibility of the semicircular arc-like structure was enhanced in the grid size containing 150,553 and 376,251 pixels than the other two grid sizes (12,221 and 96,681 grids). Hilbert-transformed T-SAFT reconstructed images for different grids are shown in Figure 7b,d,f,h. In both reconstructions (raw time traces and Hilbert transform), the defect (semicircular arc) is visible in the grid sizes containing 150,553 and 376,251 pixels.

### 3.3. Imaging in Ablation Regime

#### 3.3.1. Hole of 10 mm Diameter

**Figure 8:**
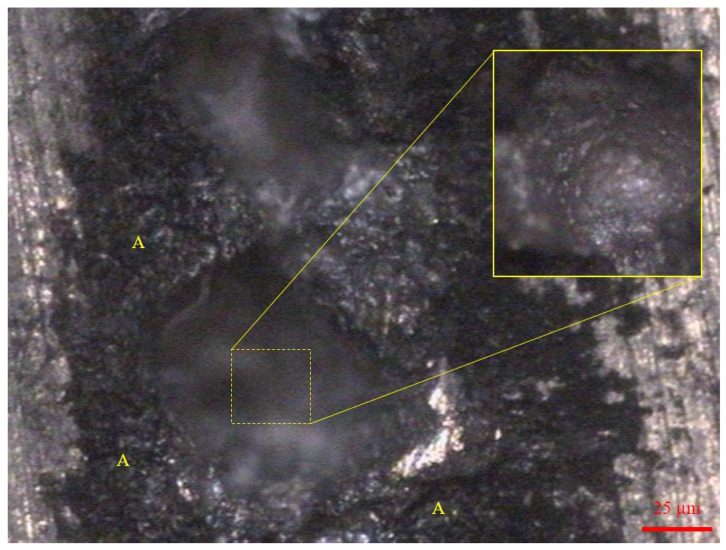
Optical microscopic image of the crater, which was formed after the ablation process. Inset: Crater depth. The regions marked with A represent the plasma plume regions.

The image reconstruction process in the T-SAFT approach solely depends on the number of elements and the amplitude of A-scan data obtained from the phased-array elements. Hence, the resolution of the inspected images can be improved if the A-scan data contain higher amplitudes or by increasing the number of elements in the predefined aperture size. The increase in the number of elements can result in higher computational time. One of the major advantages of using laser ultrasonics is that higher-amplitude signals can be obtained by generating ultrasonic waves in the ablation regime [44]. This could be achieved by reducing the flash delay of the pulsed laser in the experiment. At reduced flash delay, a highly intense laser beam was directed toward the specimen, which resulted in the formation of a crater in the focused spot. Figure 8 shows an optical microscopic image of the crater formed in the ablation process.

**Figure 9:**
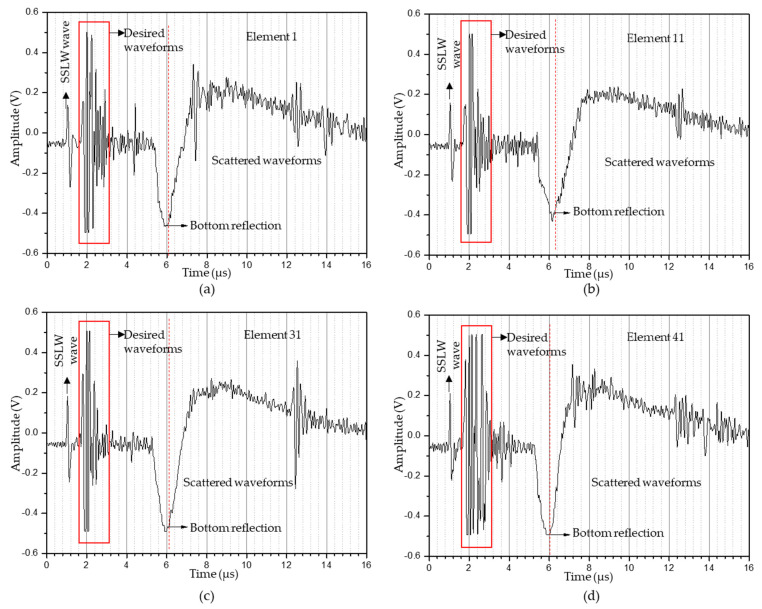
A−scan data obtained in the ablation regime (10 mm hole) from the synthesized linear array elements: (**a**) 1st element; (**b**) 11th element; (**c**) 31st element; (**d**) 41st element. The desired waveforms contain SAW wave and defect reflections. The waveforms occurring after the red dotted line (bottom reflection) were scattered waveforms (SSLW—surface skimming longitudinal wave).

**Figure 10:**
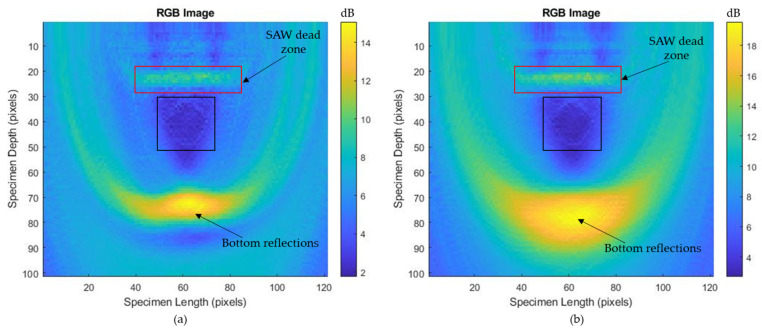

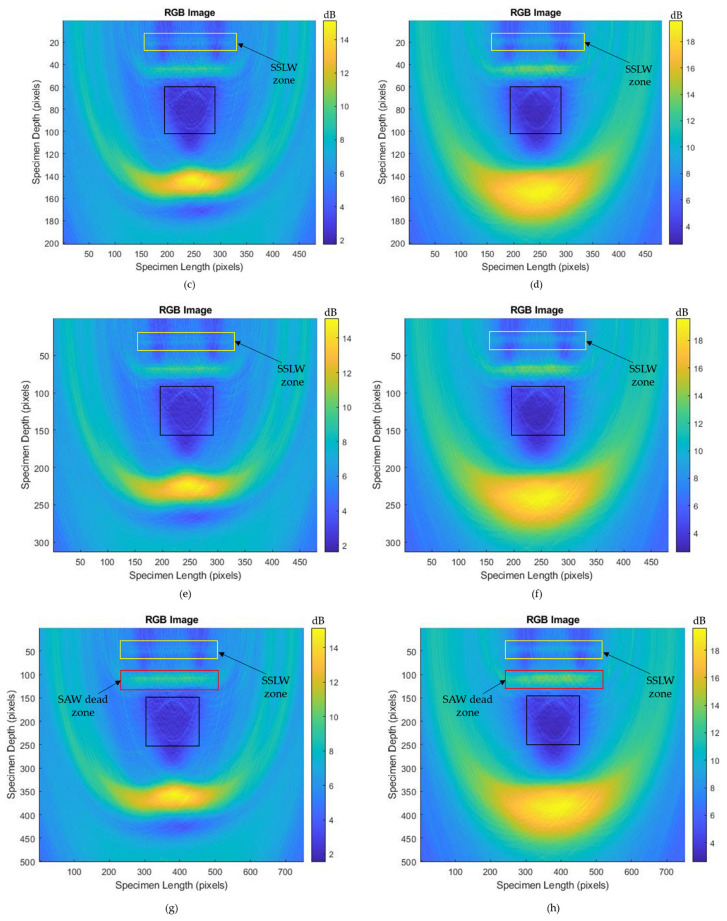
T-SAFT reconstruction images obtained in ablation regime (10 mm hole) using raw time traces: (**a**) 12,221 grids, (**c**) 96,681 grids, (**e**) 150,553 grids, (**g**) 376,251 grids; and using Hilbert-transformed data: (**b**) 12,221 grids, (**d**) 96,681 grids, (**f**) 150,553 grids, (**h**) 376,251 grids. The black box represents the defect. The yellow box represents the SSLW zone, whereas the red box represents the SAW dead zone due to the separation distance of 5 mm between the pulsed laser and LDV. Laser ablation paved the way to obtain higher-amplitude signals, which increased the pixel intensity of all grids compared to the thermoelastic regime.

The experimental parameters for imaging the 10 mm diameter hole under the ablation regime are listed in Table 3. The flash delay was reduced to 320 μs but the sample rate, number of samples, and cycle rate remained same. The captured A-scan data in the ablation regime from 1st, 11th, 31st, and 41st elements are shown in Figure 9a–d. When comparing the A-scan data obtained in the thermoelastic and ablation regimes, the ablation regime’s A-scan data showed signals with higher amplitudes than those from the thermoelastic regime. Also, the bottom reflection occurred at around 6 μs, which could be seen in the reconstructed T-SAFT images. After obtaining 41 A-scan data in the ablation regime, T-SAFT reconstructions were conducted with the raw time traces and Hilbert-transformed data. Figure 10a,c,e,g depict the T-SAFT reconstructed images made using raw time traces at various grid sizes. In Figure 10a, the defect reflections can be seen more clearly than when compared to Figure 7a. Also, by increasing the number of pixels in the region of interest, the defect reflections could be visualized more clearly when compared to the thermoelastic T-SAFT reconstruction images. Figure 10b,d,f,h represent the Hilbert-transformed reconstruction images. On comparing Figure 7g,h with Figure 10g,h, better reflections can be seen in Figure 10g,h, which is due to the higher-amplitude signal obtained in the ablation regime. Moreover, in the ablation regime, the top and bottom surfaces of the 10 mm hole were visible but, in the thermoelastic regime, only the top surface of the 8 mm hole was visible. Thus, it can be confirmed that the ablation regime improves the resolution of the inspected images by generating higher-amplitude ultrasonic waves than in the thermoelastic regime.

#### 3.3.2. Hole of 2 mm Diameter

The proposed system was effective for imaging the large defects (8 mm and 10 mm diameter) in both regimes, but the ablation regime had better defect reconstruction images due to the higher-amplitude signals. The defects in the blocks first develop at a small size and, over the long term, they grow larger, which results in catastrophic failures. Hence, it is better to detect the defects at earlier stages to avoid failures. Here, three 2 mm holes were introduced to ensure that the proposed model could detect small defects. Out of these three holes, one hole lay in the center of the aperture, whereas the other two holes lay at the two ends of the aperture. The A-scan data were recorded in the ablation regime with an aperture size of 21.5 mm containing 43 elements. The experimental parameters used for imaging the 2 mm hole are tabulated in Table 4.

**Figure 11:**
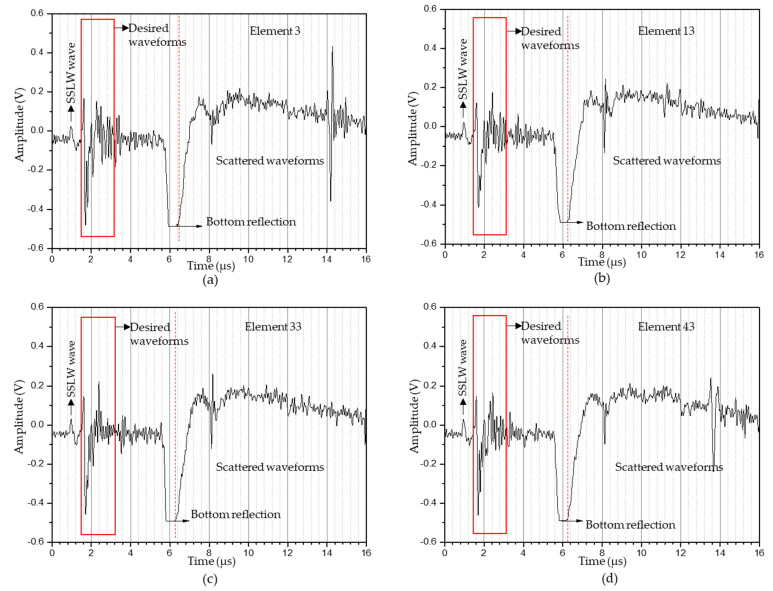
A−scan data obtained in ablation regime (2 mm hole) from the synthesized linear array elements: (**a**) 3rd element; (**b**) 13th element; (**c**) 33rd element; (**d**) 43rd element. The desired waveforms contain SAW wave and defect reflections. The waveforms occurring after the red dotted line (bottom reflection) were scattered waveforms (SSLW—surface skimming longitudinal wave).

**Figure 12:**
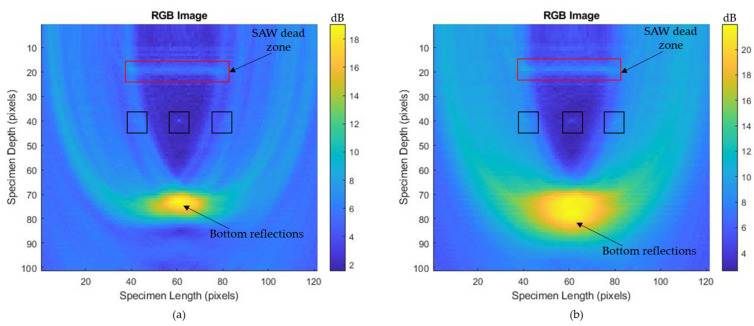

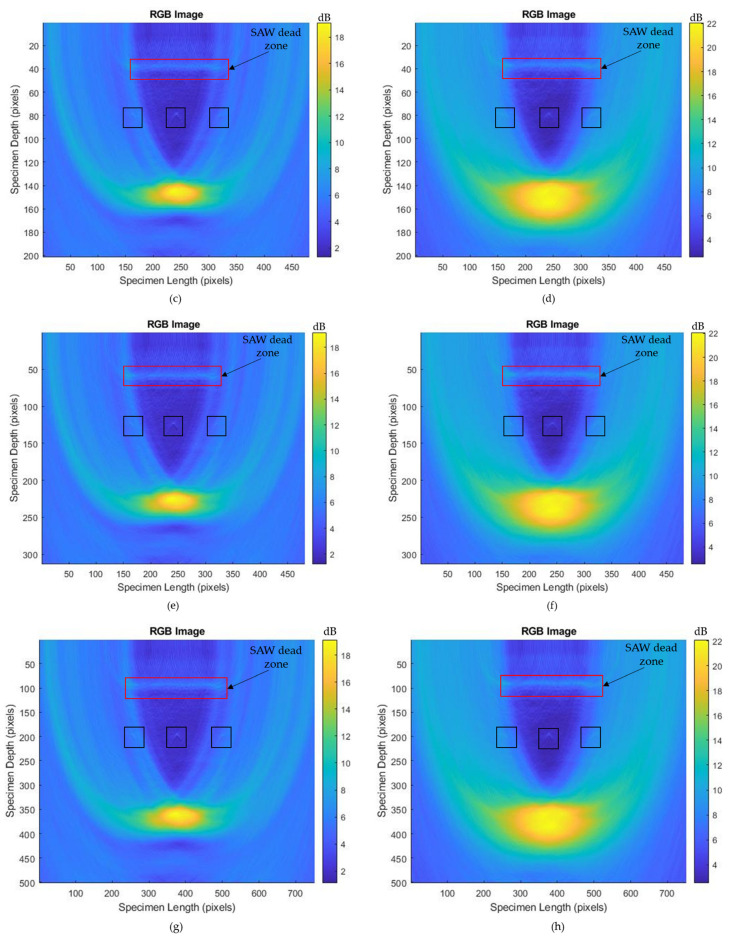
T-SAFT reconstruction images obtained in ablation regime (2 mm hole) using the raw time traces: (**a**) 12,221 grids, (**c**) 96,681 grids, (**e**) 150,553 grids, (**g**) 376,251 grids; and using the Hilbert-transformed data: (**b**) 12,221 grids, (**d**) 96,681 grids, (**f**) 150,553 grids, (**h**) 376,251 grids. The black box represents the defects. The red box represents the SAW dead zone due to the separation distance of 5 mm. The 2 mm defect hole shows minute reflections when compared with the 8 mm and 10 mm holes. These minute reflections can be seen in the grid size, containing a higher number of grids (**e**–**h**).

**Figure 13:**
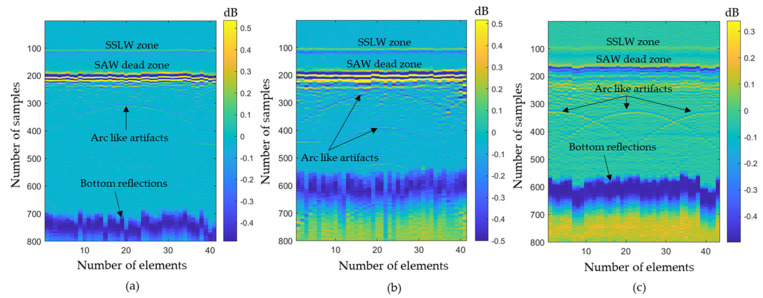
B−scan images of (**a**) 8 mm hole; (**b**) 10 mm hole; (**c**) 2 mm holes. Arc-like artifacts represent the reflected signals from the defects. SSLW zone occurs at 100 samples and SAW dead zone occurs at 200 samples. Three arc-like artifacts in (**c**) represent the presence of three 2 mm holes.

Figure 11a–d represent the A-scan data obtained from the 3rd, 13th, 33rd, and 43rd elements. Here also, the bottom reflection occurs around 6 μs. The A-scan data obtained from the 43 elements were processed via the T-SAFT algorithm to generate the inspection images. The reconstructed images obtained using the raw time traces were shown in Figure 12a,c,e,g. Since the defect reflections obtained from 2 mm hole were less compared to 8 and 10 mm holes, the defect was not visible in the grid size containing 12,221 and 96,681 pixels. On further improving the grid size from 96,681 pixels to 376,251 pixels, the visibility of the defect were better compared to the reflections obtained in the reconstructed image containing 12,221 grids. Moreover, the hole that lies in the center of the aperture has better visibility when compared with the holes that lie on both ends of the aperture. On the other hand, the reconstructed images obtained using the Hilbert-transformed data were shown in Figure 12b,d,f,h. Here also, the grid size containing higher number of pixels (376,251) has better visibility to defects than other grid sizes. But on comparing the raw time traces and Hilbert-transformed images, the images reconstructed using the raw time traces have better visibility than the Hilbert-transformed images. Figure 13a–c represents the B-scan image of 8 mm, 10 mm, and 2 mm holes. On comparing Figure 13a with Figure 13b,c, bottom reflections come earlier in Figure 13b,c than in Figure 13a. Moreover, this earlier arrival of bottom reflection does not affect the SSLW and SAW wave arrival times because, in Figure 13a–c, SSLW and SAW wave zones occur around 100 and 200 samples respectively.

## 4. Discussion

The received A-scan data comprised the surface skimming longitudinal wave (SSLW), desired waveforms (high-amplitude surface acoustic waves (SAWs) that are reflected from the edges of the sample and defect reflections), and bottom reflections. These A-scan data were used to generate a B-scan, as in Figure 13. In this figure, the SSLW wave creates a less intense zone (around 100 samples) and the SAW wave is responsible for creating a highly intense zone (around 200 samples) followed by defect (arc-like artifacts) and bottom reflections.

In the generated SAFT inspection images, a less intense SSLW zone is visible around 2.5 mm at the larger grid sizes (96,681, 150,553, and 376,251 grids) of the 8 and 10 mm holes. In the case of the 2 mm holes, the obtained A-scan data contain a low-amplitude SSLW wave. Hence, the SSLW zone is not clearly visible in the SAFT images of the 2 mm hole. The highly intense SAW dead zone (around 5 mm) is visible after the SSLW zone in most grid sizes of the 8 mm, 10 mm, and 2 mm holes. This highly intense SAW dead zone is usually visible at the top of the inspection images, where the generation and detection elements are not separated [45]. But in our case, the generation and detection beams were separated by 5 mm along the y-axis, and the SAW dead zone occurs at around 5 mm in the T-SAFT reconstructed images. As mentioned, the SAW dead zone is unavoidable in the B-scan and SAFT images. In the smaller grid sizes (12,221 and 96,681 grids), the defect reflection is not clearly visible, which might be due to the influence of this SAW dead zone. The visibility of defects is better in the 376,251 grid size amidst the effect of this SAW dead zone.

The T-SAFT image reconstruction process solely depends on the amplitude in the acquired signals. In the thermoelastic regime, the bottom reflections in the acquired signals occur around 7 µs. These reflections can be observed in Figure 7 as the dark yellow region. On the other hand, in the ablation regime, the bottom reflection occurs around 6 µs. This earlier arrival resulted in shifting of the dark yellow region, as in Figure 10. On comparing Figure 7g and Figure 10g, in the thermoelastic regime, the defect is visualized to some extent due to the lower-amplitude A-scan data acquired by the synthesized linear array elements. On the other hand, much enhanced (higher amplitude) longitudinal waves were obtained in the ablation regime [46,47]. These amplitudes improved the pixel intensity of every grid in the region of interest, thereby providing better visibility, as in Figure 10g when compared with Figure 7g.

**Figure 14:**
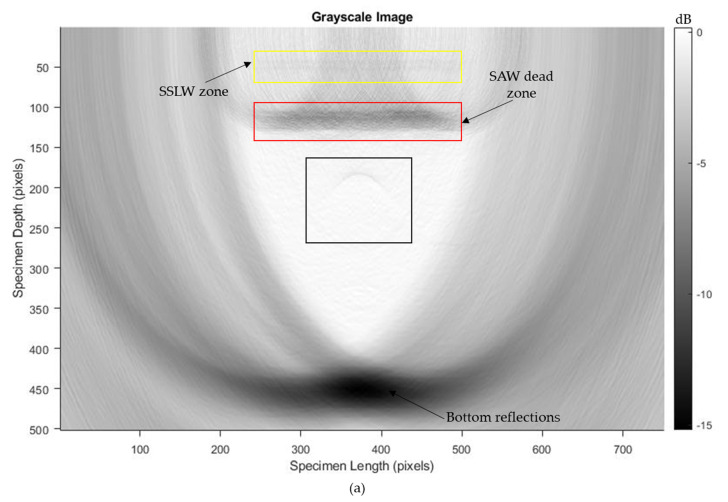

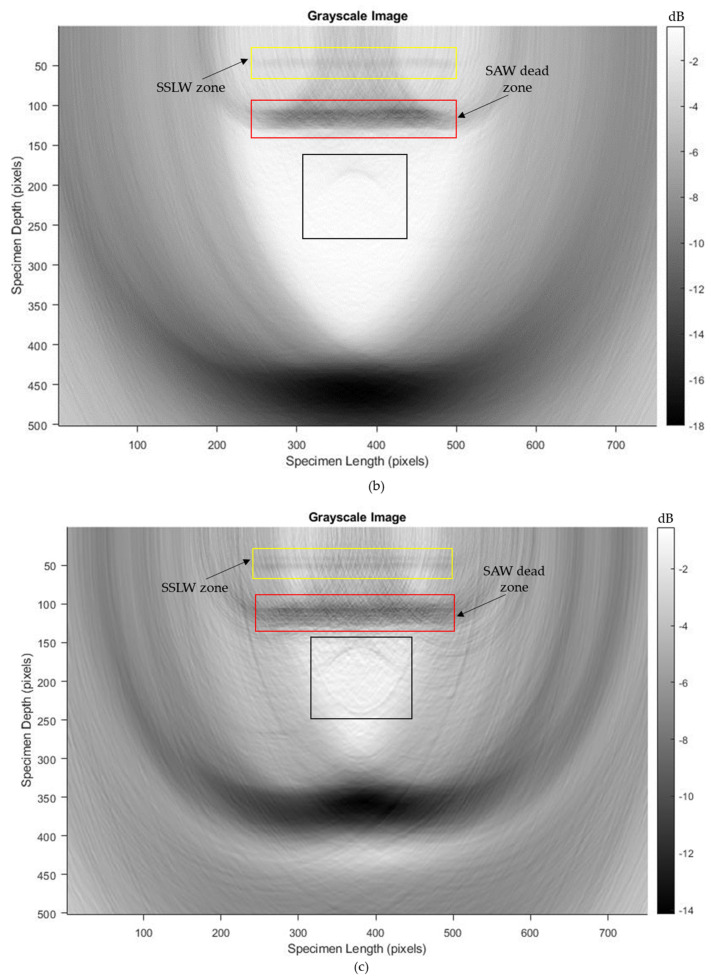

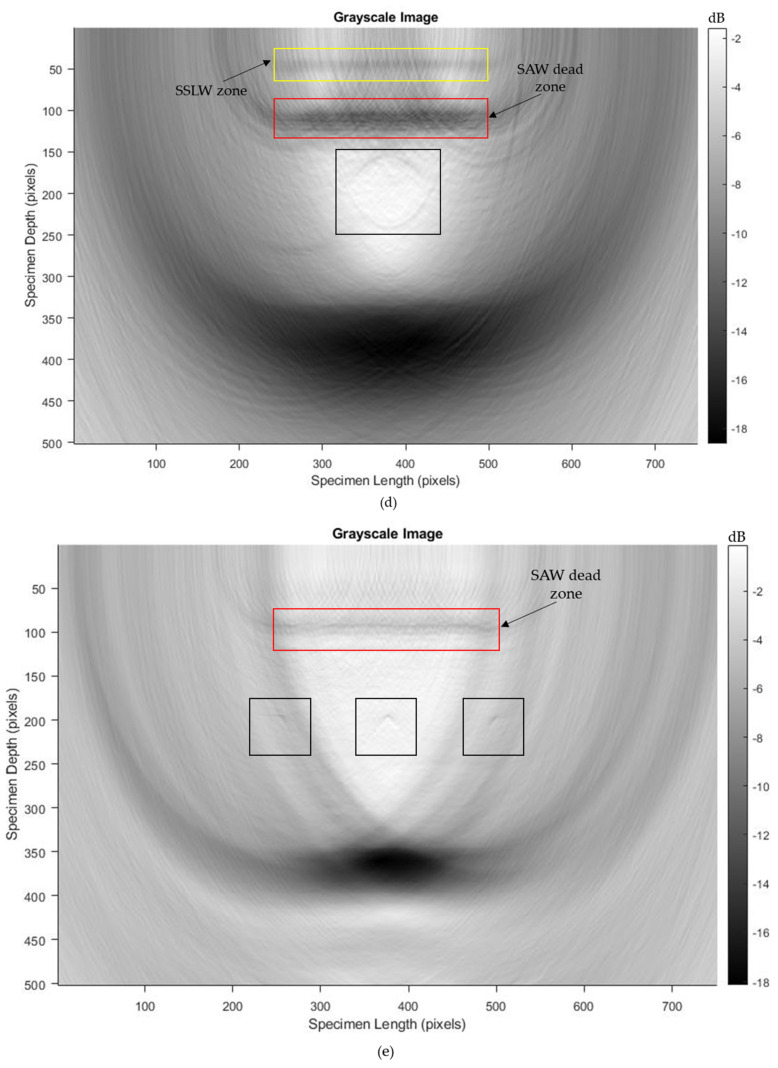

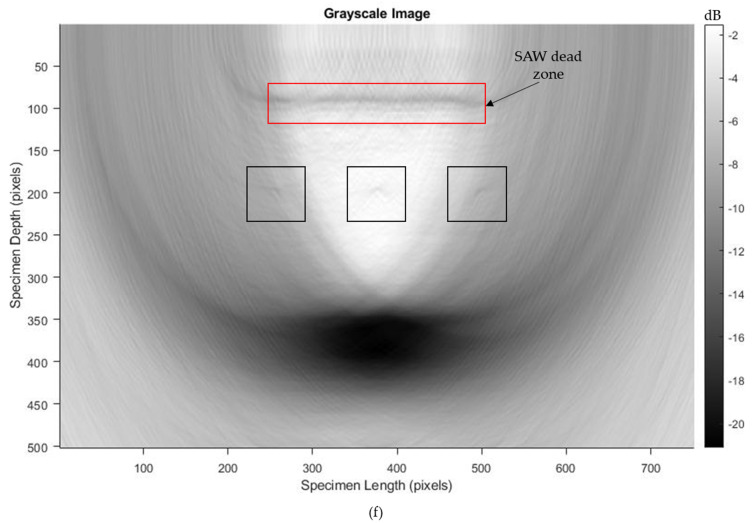
T−SAFT reconstructed images obtained in grayscale at the larger grid size (376,251 grids); (**a**,**c**,**e**) reconstructions made by using the raw time traces of 8 mm, 10 mm, and 2 mm holes; (**b**,**d**,**f**) reconstructions made by using the Hilbert−transformed data of 8 mm, 10 mm, and 2 mm holes. Black box represents the defects.

Traditional SAFT inspections do not use the Hilbert transform. However, the Hilbert transform was implemented in the phased-array probe testing. Here, both raw time traces and Hilbert-transformed data were used for the T-SAFT image reconstruction process. The Hilbert transform aids in extracting the signal’s envelope, which results in enhancing the pixel intensities of each grid in all combinations. On comparing the inspection images obtained by using raw time domain data and Hilbert-transformed data, Hilbert-transformed inspection images are dominated by the bottom reflections. The bottom reflections are more intense when compared with the inspection images obtained using the raw time-domain data. As reported, the Hilbert envelope increased the pixel intensity of each grid, thereby providing better visibility of the SSLW zone, SAW dead zone, and bottom reflection but, on the other hand, the highly intense bottom reflection might have overwhelmed the defect reflections. Hence, the defect reflections were suppressed in Hilbert-transformed inspection images due to the overwhelmed (more intense) bottom reflections. Therefore, the reconstructed images obtained using the raw time traces had better visibility of defects than the Hilbert-transformed reconstructed images. For better understanding, the grayscale images obtained at a larger grid size containing 376,251 pixels with the pixel resolution x-axis—0.08 mm/pixel and z-axis—0.05 mm/pixel are shown in Figure 14a–f. On comparing Figure 14a with Figure 14b, the defect reflection appears darker in Figure 14a than in Figure 14b. This trend is also shown in the T-SAFT reconstructed images (Figure 14d,f) of other holes. This is because the highly intense bottom reflection occurring in Hilbert-transformed inspection images might have suppressed the defect reflections.

**Figure 15:**
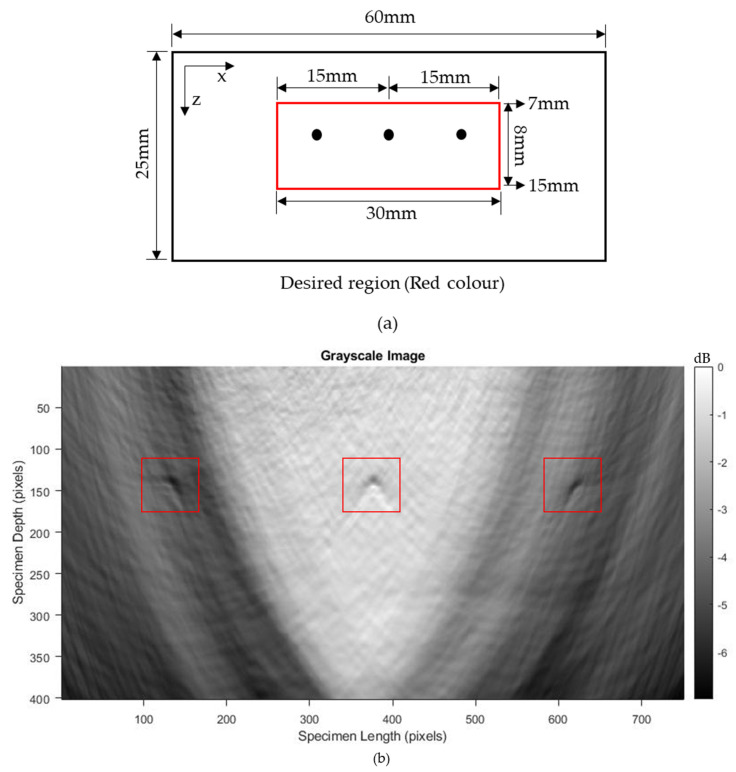

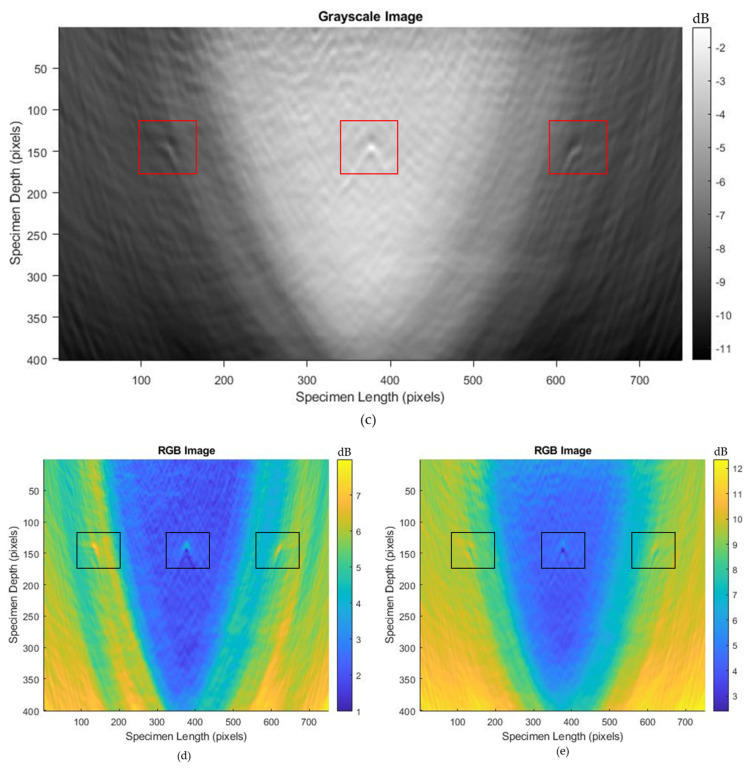
(**a**) Desired region of interest. Black dots represent the three 2 mm holes; grayscale images of (**b**) raw A−scan data and (**c**) Hilbert−transformed A−scan data; RGB images of (**d**) raw A−scan data and (**e**) Hilbert−transformed A−scan data. Red, black boxes in (**b**–**e**) represent the defects.

For imaging the three 2 mm holes with better visibility, T-SAFT reconstructions were conducted in the desired region of interest, as shown in Figure 15a. The desired region of interest was computed by considering it to be 30 mm in length (15 mm from both sides of the center hole) and with a thickness of 8 mm (from 7 mm to 15 mm of the test specimen). The desired region was divided into 301,151 grids with the resolution of x-axis—0.04 mm/pixel and z-axis—0.02 mm/pixel. Figure 15b,c represent the grayscale image reconstructions for raw A-scan data and Hilbert-transformed A-scan data, and the corresponding RGB images are shown in Figure 15d,e. On comparing the reconstructed images made using raw and Hilbert-transformed A-scan data, it can be seen that the raw A-scan data (Figure 15b) provide better visibility of the three 2 mm holes than the Hilbert-transformed A-scan data (Figure 15c).

## 5. Conclusions

The time-domain synthetic aperture focusing technique (T-SAFT) is a single-probe (contact) technique used in traditional ultrasonic testing. This technique provides better visibility of defects compared to other methods. Here, a fully noncontact system was proposed to image subsurface defects in an aluminum block with the aid of optics. The pulsed laser beam was employed to generate ultrasonic waves and the reception was ensured using a laser Doppler vibrometer. These two beams were focused on the test specimen and separated by 5 mm to minimize the effect of plasma, which was produced during the generation of ultrasonic waves in the ablation regime. By moving the test specimen with a step size of 0.5 mm, a 1D laser-induced linear phased array was synthesized, and the T-SAFT algorithm was adapted to the A-scan data obtained from the laser-induced linear phased array. The major conclusions are as follows:

(i) In the thermoelastic regime (8 mm hole), A-scan signals with lower amplitudes were obtained, which was observed in the reconstructed T-SAFT images.
(ii) The visibility of the defects was further improved in the ablation regime (10 mm hole). In this regime, much enhanced longitudinal waves (higher-amplitude signals) were obtained. These higher-amplitude signals increased the pixel intensity of each grid, thereby providing better visibility.
(iii) The ablation regime is effective for imaging small defects (2 mm hole). Minute reflections from the defects could be seen in the reconstructed images. The hole in the center of the aperture had better visibility than other two 2 mm holes.
(iv) The 5 mm distance between the pulsed laser and the laser Doppler vibrometer was observed in the reconstruction process as the SAW dead zone in all the grid sizes.
(v) Hilbert transform aids in extracting the signal’s envelope, which improved the pixel intensity values of each grid in all grid sizes. The Hilbert-transformed inspection images were dominated by highly intense bottom reflections. These reflections might have suppressed the defect reflections in the inspection images, thereby reducing the visibility of the defects.
(vi) Compared to the thermoelastic regime, the ablation regime is more effective for imaging defects, with the grid size having lateral and vertical resolutions of 0.08 mm/pixel and 0.05 mm/pixel. For small defects, the T-SAFT reconstructions at the desired region of interest had better visibility.

Thus, by adapting the T-SAFT, the proposed noncontact system can detect defects with the minimum number of elements.

## Figures and Tables

**Table 1:** Mechanical properties of the aluminum block (AA 6082 T6).

Property	Value
Young’s modulus	71 GPa
Poisson’s ratio	0.33
Density	2.71 g/cm^3^
Dimension	60 × 60 × 25 mm

**Table 2:** Experimental parameters in the thermoelastic regime (8 mm hole).

Parameter	Value
Cycle rate	5 Hz
Flash delay	360 µs
Aperture size	20.5 mm
Aperture elements	41 elements
Number of samples	1600
Sample rate	100 MHz

**Table 3:** Experimental parameters in ablation regime (10 mm hole).

Parameter	Value
Cycle rate	5 Hz
Flash delay	320 µs
Aperture size	20.5 mm
Aperture elements	41 elements
Number of samples	1600
Sample rate	100 MHz

**Table 4:** Experimental parameters in ablation regime (2 mm hole).

Parameter	Value
Cycle rate	5 Hz
Flash delay	320 µs
Aperture size	21.5 mm
Aperture elements	43 elements
Number of samples	1600
Sample rate	100 MHz

## Data Availability

The experimental datasets obtained from this research work and the analyzed results are available from the corresponding author on reasonable request.
